# CoViD-19 effects on social-emotional development: Putative underlying mechanisms

**DOI:** 10.6026/9732063001987

**Published:** 2023-10-31

**Authors:** Rodriguez-Monge Michelle, Chiappelli Francesco

**Affiliations:** 1 Costa Rican Institute of Clinical Research, San José, Costa Rica; Stars Therapy Services, San Diego, CA 91913; 2 Dental Group of Sherman Oaks, CA 91403, USA); 3 Center for the Health Sciences, UCLA, Los Angeles, CA; 90095

**Keywords:** CoViD-19, Critical Period hypothesis, Vygotsky's zone of proximal development, Piaget's developmental stages, Bowlby's attachment theory

## Abstract

Early childhood is the timely and critical period in the growth of the human being when the trajectory of children's holistic development is traced, and the foundation for their future as well-established and productive adults is set. The CoViD-19 pandemic produced profound changes in everyday life almost everywhere in the world. The personal, social and societal restrictions imposed during the CoViD-19 pandemic unquestionably blunted early childhood development by depriving young children from normal and healthy attachments through secure relationships with parents, teachers and peers. Furthermore, the public health measures enacted to counter the spread of the pandemic (e.g., mandatory masking, lockdown) contributed to a lack of social interactions essential for childhood development, and provoked perceptions of psycho-emotional stress (e.g., objective fear of the masked interlocutor, perceived fear of abandonment) in the children, which may have hampered critical periods of development. Based on theoretical foundation and our observations in the field, we propose that early intervention support may have a significant impact on the development of children victims of the effect of the CoViD-19 pandemic.

## Background:

The Corona Virus Disease of 2019 (CoViD-19) pandemic produced over 770 million cases and 7 million deaths worldwide in less than four years. Since its onset in late 2019/early 2020, it has created a major public health catastrophe across all countries globally. It has indiscriminately taken children and elderly, mothers, daughters and sisters, fathers, brothers and sons of all ages, occupations, ethnicities, and nationalities. Even among its survivors, it has impacted long-term the health of its victims [1]. CoViD-19 has disrupted social communities and families. A recent systematic review showed that anxiety, depression, disturbances in sleep and appetite, and impairment in social interactions are the most common sequelae of the pandemic among children and adolescents [2]. We discussed evidence that confirms and expands the impact of the CoViD-19 pandemic in neonatal and early childhood social, emotional, and cognitive development. We proposed that the public health measures enacted to counter the spread of the pandemic (e.g., mandatory masking, lockdown) contributed to a lack of social interactions essential for childhood development, and provoked perceptions of psycho-emotional stress (e.g., objective fear of the masked interlocutor, perceived fear of abandonment) in the children, which may have hampered critical periods of development (cf., Penfield's & Robert's 1959 critical sensitive period hypothesis of development). We proposed that early intervention services could, and should be optimized to fill in the gaps in development procured by the pandemic, and to allow essential social, emotional, and cognitive skills to develop appropriately [3]. It is possible and even probable that the underlying mechanisms responsible for the delay in social, emotional and cognitive skills development and in some cases the regression in development of these skills, among young children in families disrupted by the pandemic may involve deep psychological substrata. It follows that the targeted early intervention programs, which, we proposed [3], could counter the pandemic-derived delays in the children's acquisition of appropriate social and emotional abilities, ought to be grounded in sound early childhood and developmental psychology models and theories. To be clear, early childhood is the timely and critical period in the growth of the human being when the trajectory of children's holistic development is traced, and the foundation for their future as well-established and productive adults is set. For children to achieve their full potential, they need health care and nutrition, protection from harm, opportunities for early learning, responsive caregiving, and a sense of security. This proposition was first articulated in our contemporary times by Lev Vygotsky (1896-1934), who posited that children develop, create and shape mental and cognitive abilities through language and by interacting directly with, and thus constructing for themselves the cultural and social environment they need. Vygotsky also emphasized the essential role of play and role-modeling in early childhood development, activities that were significantly altered during the pandemic [4]. In brief, and to use Vygotsky's term, the CoViD-19 pandemic reduced the children's zone of proximal development, the psycho-emotional, cognitive, and social range, or space where the child is able to perform with support from a parent, teacher or peer with more knowledge or expertise, and beyond which the learner cannot even with support and guidance.

Vygotsky agreed with some, but not all the propositions in Jean Piaget's (1896-1980) theory of early child development [5]. Piaget defended that early childhood development was first and foremost driven by the process of biological development of the child. In his view, early childhood psychological development involved a transition driven by an unspoken yet clearly perceived by the child "spontaneous conviction" from egocentrism to sociocentrism. He observed that early childhood development proceeded as a gradual progression from intuitive and self-focused to better reasoned and socially acceptable responses. Piaget argued that the process was entirely dependent upon the social interaction and challenge to which children were exposed [5]. In brief, Piaget described four critical developmental stages, and argued that progression through these levels of developments depended upon the constructivist concepts of learning, (i.e., générative learning), assimilation, (i.e., interpreting new information within the framework of existing knowledge), and accommodation (i.e., adapting one's knowledge base to cope with things that do not fit the existing frameworks). Piaget described the sensorimotor stage (birth to age roughly two), where children experience the world through movement, their senses and their own views and perceptions. The pre-operational stage (about when the child begins to speak to about age of seven), he proposed, is the period when social, emotional, and cognitive development begin to emerge, although children still have trouble seeing things from different points of view than theirs. It is during the pre-operational Symbolic Function Substage (age about 2 to about 4) that children begin to comprehend the use of symbols to represent reality, and to translate their perceptions about physical models of the world around them into symbols [5,6]. It is at that stage that social constraints imposed by the CoViD-19 pandemic, such as masking of parents, teachers and peers, lockdown and deprivation of social interactions and said physical models of the world around them could most dramatically impair pre-operational simulation and accommodation. In addition, during the pre-operational Intuitive Thought Substage (age about 4 to about 7) children become curious, ask questions, and process their observations, perceptions and the answers they receive in a form of primitive reasoning to interpret the world around them [5, 6]. It is self-intuitive that if the world they observe around them is a world of masked individuals, restricted social interactions, and lockdown, their intuitive thought development will be severely impaired.

Another critical variable in early childhood development is attachment. In that context, John Bowlby (1907-1990) proposed the concept of the "secure base" as critical for early childhood development [7]. He defended that children develop an internal working model of the self (i.e., a self-model) and an internal working model of others (i.e., the other-model). These models originate from early experiences with parents, peers and teachers, and shape their current and future interpersonal relationships (i.e., sociability, avoidance, loneliness), as well as views and identity of self (i.e., self-confidence, self-esteem, dependency). The infant and the young child have an almost insatiable need for a secure relationship with parents, peers, older siblings, and teachers, without which, Bowlby's theory emphasizes, normal social and emotional development cannot and will not occur [7]. To be clear, the personal, social, and societal restrictions imposed during the CoViD-19 pandemic are unquestionably limited at best, and deprived young children from developing normal and healthy attachments through secure relationships with parents, teachers, and peers. These theoretical foundations of early childhood development, taken together with observations that stages of early childhood development are much broader temporally then the early motor, social, and language developmental milestones [8], proffer a putative construct of mechanisms for the observed delays in early childhood, childhood, and adolescent psycho-emotional, social and cognitive development [1] [2]. They suggest that the perceived experience of attachment to parents and siblings on the part of children (age 0 to 3; Piaget's sensorimotor stage) and their zone of proximal (Vygotsky) were blunted, impaired, and perhaps irremediably damaged at the height of the CoViD-19 pandemic. 

## Conclusion:

In conclusion, we propose that the development of actionable early childhood intervention modules must incorporate special attention to these domains, and focus on social-emotional development and communicative development. Through timely interventions and parent education provided by early intervention services, the repercussions of the CoViD-19 pandemic can be mitigated. It is vital for families with children experiencing developmental delays of any kind to address such in an effective manner. Through early intervention services, children and families who experienced the CoViD-19 pandemic and are experiencing developmental delays will receive potentially vital support and guidance on how to fill in the gaps of development produced by this life changing unprecedented period.
